# Influence of the Planning Parameters of a New Algorithm on the Dosimetric Quality, Beam-On Time and Delivery Accuracy of Tomotherapy Plans

**DOI:** 10.3390/cancers16101883

**Published:** 2024-05-15

**Authors:** Théo Burckbuchler, Nicolas Dehaynin, Claudine Niederst, Laurent Bartolucci, Halima Elazhar, Delphine Jarnet, Florence Arbor, Philippe Meyer

**Affiliations:** 1 Medical Physics Unit, Institut de Cancerologie de Strasbourg (ICANS), 17 Rue Albert Calmette, 67200 Strasbourg, France; 2 Team IMAGeS, ICUBE Laboratory, University of Strasbourg, CNRS, UMR 7357, 67412 Illkirch, France

**Keywords:** tomotherapy planning, VOLO Ultra, delivery quality assurance, radiotherapy

## Abstract

**Simple Summary:**

A new planning and optimization algorithm, VOLO^TM^ Ultra, has been introduced for tomotherapy planning. This algorithm includes new features that significantly changed how treatments are planned and optimized. We have reoptimized 1056 treatment plans for 36 patients and six anatomical locations (breast with and without nodes, head and neck, cervix, esophagus, and hypofractionated). The results allowed us to characterize the impact of the accelerated treatment, leaf open/close time cutoff, normal tissue objective weight, number of optimization rounds, and use of rings on dosimetric quality and beam on time. The aim was to give a better understanding of the impact of the planning parameters on tomotherapy treatments to improve the quality of treatment for patients undergoing tomotherapy.

**Abstract:**

Background: This work aimed to determine the optimum VOLO^TM^ Ultra algorithm parameters for tomotherapy treatments. Methods: 1056 treatment plans were generated with VOLO^TM^ Ultra for 36 patients and six anatomical locations. The impact of varying four parameters was studied: the accelerated treatment (AT), leaf open/close time (LOT) cutoff, normal tissue objective (NTO) weight, and number of iterations. The beam-on time and dosimetric metrics were quantified for the target volumes and organs at risk (OARs). Delivery quality assurance measurements were obtained for 36 plans to assess the delivery accuracy. Results: The mean beam-on time for the helical tomotherapy and TomoDirect (TD) plans decreased by 26.6 ± 2.8% and 17.4 ± 4.3%, respectively, when the accelerated treatment parameter was increased from 0 to 10, at the expense of the planning target volume (PTV) coverage (2% lower D_98%_) and OAR dose (up to 15% increase). For TD plans, it seems preferable to systematically use an AT value of 10. Increasing the number of iterations beyond six seems unnecessary. In this study, an NTO weight of approximately 10 appears to be ideal and eliminates the need to use rings in the treatment plan. Finally, no correlation was found between the leaf open/close time cutoff and the delivery accuracy, while a leaf open/close cutoff of 60 ms seemed to degrade dosimetry quality. Conclusion: Optimal values for the AT, LOT cutoff, NTO weight, and number of optimization rounds were identified and should help improve the management of patients whose tomotherapy treatments are planned with VOLO^TM^ Ultra.

## 1. Introduction

Since the first patient was treated with helical tomotherapy in 2002 [1], technical innovations have led to improvements in the quality of patient care, including dynamic jaws [2], real-time motion tracking [3], and helical kVCT [4]. Treatment planning system (TPS) algorithms have considerably evolved over the last twenty years, with several tomotherapy TPSs, including the cluster upgrade from a central processing unit (CPU) to a graphic processing unit (GPU) in 2012 [5], referred to as voxel-less optimization (VOLO^TM^). In 2018, the tomotherapy TPS was replaced by the precision TPS (Accuray, Sunnyvale, CA, USA), which was improved in 2021 with the integration of VOLO^TM^ Ultra, a new planning and optimization algorithm [6].

VOLO^TM^ Ultra includes a new L-BFGS-B-based optimization algorithm [7], with faster convergence to the optimal solution than the previous VOLO^TM^ tool (still available as VOLO^TM^ Classic in the precision TPS). The initial sinogram, which is used to initialize the optimizer, is calculated using the same algorithm as VOLO^TM^ Classic. The VOLO^TM^ Ultra optimizer dose calculations are performed with a fluence-convolved broad-beam (FCBB) algorithm in each iteration and a more precise cone convolution superposition (CCCS) algorithm every 20 iterations, which constitutes an optimization round. Due to the fast convergence characteristics of the L-BFGS-B algorithm, the default number of rounds is set to three by the software vendor (version 3.3.1.2).

The VOLO^TM^ Ultra algorithm also includes new features that significantly changed how treatments are planned and optimized. These new tools include a new normal tissue objective (NTO) tool that allows better control of the dose in regions outside delineated structures and does not involve the use of optimized structures such as rings. Several parameters of the NTO tool can be modified by the user, including the weight and other elements in the cost function. To control the beam-on time (BOT), the VOLO^TM^ Ultra algorithm includes a new parameter called the accelerated treatment (AT), which replaces the modulation factor (MF) in the previous VOLO^TM^ algorithm. Compared to the MF, the AT parameter improves efficiency by limiting the longest and shortest leaf opening times, which greatly reduces the treatment time while having little impact on the dosimetric quality of the plan [8]. Another new parameter in the VOLO^TM^ Ultra algorithm is the leaf open/close time (LOT) cutoff: the leaf remains open or closed when the open or close time is less than the cutoff. Increasing this parameter should theoretically improve the delivery accuracy [9].

The impact of the AT, LOT cutoff, NTO, number of optimization rounds, and use of rings on dosimetric quality and BOT were characterized in this study by recalculating 1056 treatment plans. The aim was to help better understand VOLO^TM^ Ultra planning parameters behavior for tomotherapy treatments, which means first of all identifying and avoiding the parameters that produce inefficient plans, for example, those that increase treatment time without any benefit on target volume or organs at risk (OAR). A summary giving a quantitative view of the impact of varying these optimization parameters on treatment time, PTV covering, and OAR sparing is also proposed, enabling the user to choose those he deems most optimal, according to its planning priorities.

## 2. Materials and Methods

### 2.1. Patients

Patients treated between November 2022 and February 2023 at the Institut de CANcérologie de Strasbourg (ICANS, Strasbourg, France) were retrospectively selected for this study. Tomotherapy treatments for these patients were delivered on one of the department’s three machines: Tomo-HiArt (installed in 2008), Tomo HD (installed in 2013), or Radixact (installed in 2019). The study complied with the ethical rules of the hospital and is registered as IRB-2023-6 with the hospital institutional review board.

Six anatomical locations with different fractionations were chosen to reflect the majority of patients treated at ICANS with tomotherapy plans: the characteristics of the treatment plans by location are presented in Table 1. The so-called “hypofractionated” location does not include a particular anatomical area and instead groups together palliative treatment plans with 2 × 6.5 Gy fractionation. The results of the VOLO^TM^ Ultra algorithm were characterized for fractionations ranging from 1.8 Gy to 6.5 Gy per fraction. Six patients per site were selected, and a total of 36 patients were included in the study. Given its exploratory nature and the constraints involved, this number of patients was deemed sufficient to reach the study’s objectives.

### 2.2. Optimization Parameters

The 36 patients were treated with tomotherapy based on plans that were previously calculated with the VOLO^TM^ Classic algorithm. For this study, all plans were reoptimized with the VOLO^TM^ Ultra algorithm by an experienced physicist (15 years of planning experience in tomotherapy) to obtain a plan equivalent to that calculated with VOLO^TM^ Classic in terms of dosimetric quality; this plan was used as the initial plan. The field width (FW) and pitch of the plans depend on the location and are presented in Table 1. The pitch was reoptimized based on the new features, allowing us to obtain Chen’s pitch to reduce the thread effect [10,11]. In the performance tab, the accelerate optimization parameter is set to 1. The optimization grid resolution is set to low and medium for the HT and TD plans, respectively. At the end of each optimization round, a final dose with a high-resolution grid and a rescaled dose to 50% of the planning target volume (PTV) were obtained.

The optimization constraints (weights, dose, and volume) of the target volumes and OARs of these initial plans were fixed and did not vary during this study (see Appendix A for examples of the constraints used in this study). The parameters that varied included the AT, LOT cutoff, NTO (applied on the body contour), and number of rounds. Default parameters were used for the NTO curve (5 mm/100%, 20 mm/50%, and 100 mm/20%). The usefulness of applying a 5 cm ring around the PTV, which we used in our clinical practice with the VOLO^TM^ Classic algorithm to accelerate the dose fall-off and reduce the dose to normal tissue, was also assessed. The details of the parameter combinations used in this study are presented in Table 2. For each patient, the initial plan calculated with VOLO^TM^ Ultra was reoptimized with all parameter combinations. A total of 1056 plans were reoptimized: 648 plans with varying AT and LOT, 300 plans with varying NTO (with or without a 5 cm ring), and 108 plans with varying iteration rounds (see Table 2).

### 2.3. Metrics Used for Treatment Plan Quality Assessment

To characterize the quality of each of the 1056 reoptimized plans, the BOT, PTV D_98%_ (or V_95%_ for the breast treatment plans), and D_2%_ were systematically measured. For the OARs, dosimetry data specific to each location were collected (see list in Appendix B). The mean dose inside the patient contour excluding the PTV (body-PTV) was used to study the impact of the NTO feature and the 5 cm ring on the results.

### 2.4. Impact of the LOT Cutoff on the Delivery Accuracy

As previously stated, an increased LOT cutoff parameter should improve delivery accuracy [9], which means reducing the differences between the theoretical treatment plan and the one actually delivered by the machine. To assess the usefulness of this new optimization parameter, we obtained delivery quality assurance (DQA) measurements based on several plans. Given the time required to perform the measurements, we could not obtain the measurements for all 1056 plans. Thus, we chose to obtain measurements for 36 plans, focusing on the case of the whole breast with nodes, which is the most frequently treated location in our department (see Table 3). TD plans are not assessed because of the typical distribution of LOT values for TD treatments (almost no LOT < 60 ms). The plans were evaluated using a Radixact-type machine (Accuray, Madison, WI, USA) with an Arccheck (Sun Nuclear, Melbourne, FL, USA). To minimize the impact of the detector’s position on results, all measurements for a patient are performed during the same session, without moving the detector. Various local and global absolute gamma index values were recorded, with a threshold of 10%: 3%/3 mm, 2%/2 mm, 1%/1 mm, 3%/2 mm, and 2%/1 mm.

For each of these measured plans, we extracted 65 complexity metrics using the MATLAB (version R2023) program TCoMX developed by Cavinato et al. [12,13] to study the correlations among the LOT cutoff parameter, the gamma index values, and these metrics.

### 2.5. Statistical Analysis

Spearman and Pearson correlation tests were performed to establish potential links between the LOT cutoff values, gamma index values, and complexity metrics. The tests were performed in Python, version 3.11.4, using the Jupyter Notebook web application, version 7.0.0. For the Pearson and Spearman correlations, data were formatted using the Pandas library, version 2.0.3. Heatmaps were produced using the Seaborn library, version 0.12.2.

## 3. Results

The complete raw data are available in a data repository (https://data.mendeley.com/datasets/nz6ss5rgb5/draft?a=30c60a3c-b9d5-4bbc-84cc-c526a3cee215 (accessed on 1 May 2024)). The gamma index values for the 36 measured plans are provided in Appendix C.

**Figure 1:**
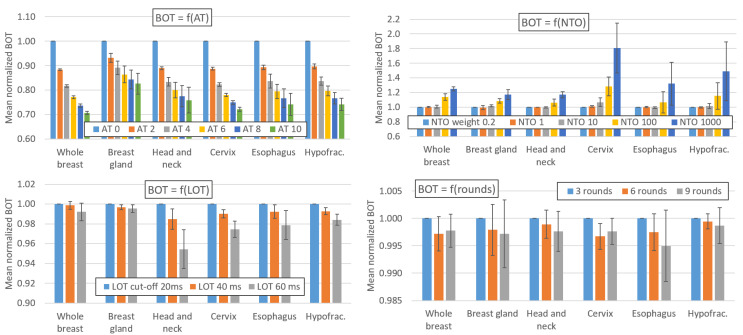
Impact of variations in the AT, LOT cutoff, NTO weight, and number of optimization rounds on BOT. Values are averaged over the six patients for each location. The AT variation values are averaged over the three LOT values, and the LOT variation values are averaged over the six AT values. The values are normalized to the first value of each parameter studied to analyze the variation in the parameters. The error bars represent one standard deviation.

**Figure 2:**
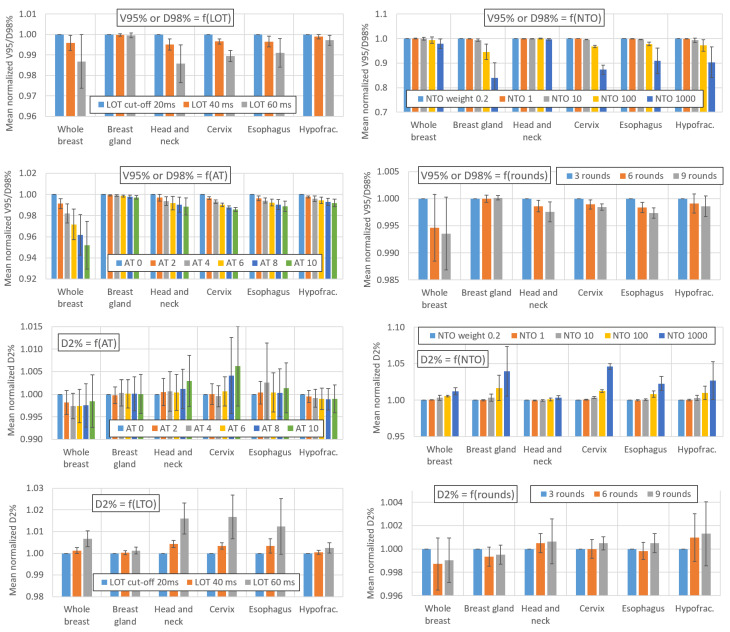
Impact of variations in the AT, LOT cutoff, NTO weight, and number of optimization rounds on the PTV dosimetric indices. Values are averaged over the six patients for each location. The AT variation values are averaged over the three LOT values, and the LOT variation values are averaged over the six AT values. The values are normalized to the first value of each parameter studied to analyze the variation in the parameters. The error bars represent one standard deviation.

**Figure 3:**
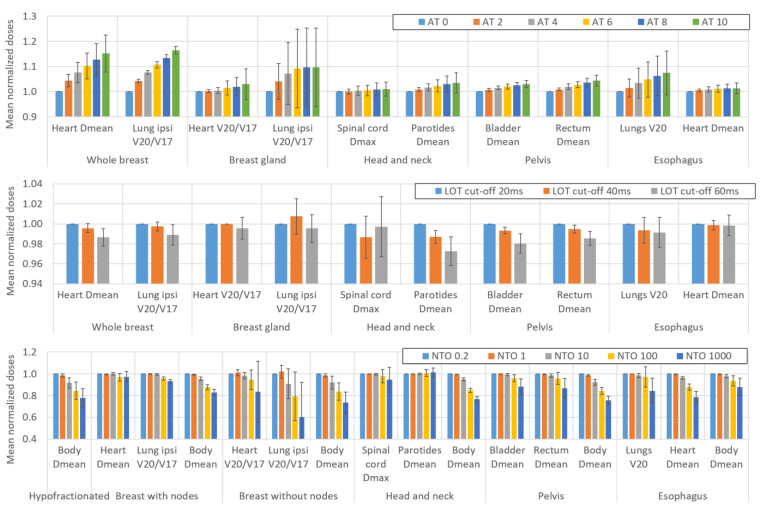
Impact of variations in the AT, LOT cutoff, and NTO weight on the OAR dosimetric indices. Not all OARs are reported for clarity. The values are averaged over the six patients for each location. The AT variation values are averaged over the three LOT values, and the LOT variation values are averaged over the six AT values. The values are normalized to the first value of each parameter studied to analyze the variation in the parameters. The error bars represent one standard deviation.

Figure 1 and Figure 2 show the impact of variations in the AT, LOT cutoff, NTO, and number of optimization rounds, respectively, on the BOT and PTV dosimetric indices. For simultaneous integrated boost (SIB) locations, only the PTV with the highest dose is considered. The impact of these parameters on the main OAR dosimetric indices is illustrated in Figure 3 (neither all the indices listed in Appendix B nor the number of optimization rounds are shown for clarity; these data can be found in the data repository and in Appendix D). Each value is the average value of the six patients for each location. The AT variation values are averaged over the three LOT values, and the LOT variation values variation values are averaged over the six AT values. The values are normalized to the first value of each studied parameter to analyze the variation in the parameters.

The impact of using the ring is shown in Appendix E, which presents the mean deviation between plans calculated with and without the 5 cm ring as a function of the NTO value for the BOT, PTV D_98%_ or V_95%_ and body-PTV dose. Only four locations were studied: whole breast, breast gland, H&N and esophagus.

Notably, the data for 12 of the 1056 reoptimized plans are not included, as they were impossible to rescale (by software design, as the doses could be rescaled by up to only 10%). These 12 plans have a common LOT cutoff of 60 ms and a large fraction of leaves with open/close times less than 60 ms before the final dose calculation. The optimizer enforces these leaf open times often, increasing the dose by more than 10% compared to the prescribed dose.

## 4. Discussion

### 4.1. Effect of the AT Parameter on the Beam-On Time

As expected, Figure 1 shows that the AT value has a major impact on the BOT. For HT treatment sites, the treatment time is reduced by 26.6 ± 2.8% when the AT is increased from 0 to 10. The influence of the AT parameter on the BOT is similar for the HT plans, regardless of the site and dose/fraction. The AT parameter has a weaker impact on the TD plans: the BOT decreases by 17.4 ± 4.3% when the AT is increased from 0 to 10. The variation in the BOT with the AT parameter is not linear and is more pronounced for low AT values. For locations treated with HT plans, the BOT decreases by 11%, 6.8%, 4.9%, 3.8%, and 3.2% on average when the AT is increased from 0 to 2, 2 to 4, 4 to 6, 6 to 8 and 8 to 10, respectively.

The relationship between the BOT and AT parameters is valid when the gantry period is greater than 11.8 s, which is the case for the patients considered in this study. However, we emphasize the importance of choosing the FW and pitch according to the location and dose/fraction to produce effective plans (i.e., for gantry periods > 11.8 s) [14].

### 4.2. Effect of the LOT, NTO and Number of Rounds on the Beam-On Time

Figure 1 shows that the impact of the NTO value on the BOT is negligible for NTO values below 10; however, this parameter has a large impact on the BOT for NTO values between 100 and 1000. The BOT increases by 0.1% and 1.6% on average when the NTO varies from 0.2 to 1 and 0.2 to 10, respectively, but it increases by 14.1% and 40.8% when the NTO increases from 0.2 to 100 and 0.2 to 1000, respectively. However, these observations, as well as those related to the impact of the NTO in the following paragraphs, are only generalizable to treatment plans for which the target weights and OAR goals used in the optimization process are comparable to those used in our study (see Appendix A).

The other optimization parameters have small impacts on the BOT. The BOT decreases by 0.8% and 2.3% on average when the LOT cutoff is increased from 20 to 40 or 20 to 60 ms, respectively. Moreover, the BOT decreases by 0.2% when the number of optimization rounds is increased from 3 to 6 and remains stable (<0.1%) when the number of rounds is increased from 6 to 9.

### 4.3. Effect of the AT, LOT, NTO and Number of Rounds on the PTV Coverage

For breast glands treated with TD plans, Figure 2 shows that the impact of the AT parameter on the PTV V_95%_ metric is negligible (<0.3% as the AT increases from 0 to 10). For locations treated with HT plans, the PTV D_98%_/V_95%_ values decrease by an average of approximately 2% when the AT is increased from 0 to 10. This decrease is more pronounced for the whole breast case, with the PTV coverage decreasing by approximately 5%. There appears to be no correlation between the PTV D_2%_ and AT values, regardless of the treatment location.

Increasing the LOT cutoff value leads to a decrease in the PTV D_98%_/V_95%_ values for locations treated with the HT technique: the decrease is small when the LOT is increased from 20 to 40 ms (mean decrease of 0.3%) and larger when the LOT is increased from 20 to 60 ms (mean decrease of 1%). For breast glands treated with TD plans, the LOT cutoff parameter had no impact on the PTV V_95%_ value. The D_2%_ value increased by 0.3% and 1% on average when the LOT was increased from 20 to 40 or 20 to 60 ms, respectively.

We note that these values are averaged and do not reflect interpatient variations, which may be significant for certain parameter configurations. For one of the whole breast patient, a LOT cutoff parameter moving from 20 to 60 s and combined with an AT value of 10 led for example to a decrease of more than 8% in the PTV V_95%_ value.

For NTO values up to 10, the impact of the NTO on the PTV D_98%_/V_95%_ and D_2%_ is negligible. Higher degradation of the PTV coverage is observed for NTO values between 100 and 1000: the PTV D_98%_/V_95%_ decreases by approximately 2% and 7%, respectively, while D_2%_ increases by approximately 1% and 2%, respectively.

The PTV D_98%_ and V_95%_ values decrease respectively by 0.2% and 0.5% when the number of optimization rounds increases from 3 to 6 and remain stable (<0.1%) when the number of optimization rounds increases from 6 to 9. The number of optimization rounds has no impact on the PTV D_2%_ value (<0.1%).

### 4.4. Effect of the AT, LOT, NTO and Number of Rounds on the Dose to the Organs at Risk

Given the diversity of the OARs and locations studied, it is difficult to quantify the average impact of the optimization parameters on the OAR dosimetric indices. We attempted to estimate the qualitative trends based on the variations in the dosimetric indices of the two main OARs for each location (shown in Figure 3). Moreover, comparisons with the variations in the dosimetric indices of the other OARs were performed to confirm the results (see data repository).

In general, Figure 3 shows that increasing the AT parameter leads to an increase in the doses delivered to the OARs. When the AT increases from 0 to 10, the increase in the OAR dosimetric indices ranges from one percent (1.4% for the mean dose to the heart for the esophagus location) to a few percent (3.5% for the mean dose to the parotids for the H&N location) and can reach up to approximately 15% (16.4% in the V_20Gy_/V_17Gy_ of the ipsilateral lung for the whole breast location).

Increasing the LOT cutoff value leads to a significant reduction in the dose delivered to the OARs. This reduction is 0.5% and 1% on average when the LOT cutoff value is increased from 20 to 40 and 20 to 60 ms, respectively. However, the standard deviations of both the LOT and AT are relatively high, leading to potentially significant disparities between different patients and various OARs.

The impact of the NTO on the OAR doses is also highly patient and location-dependent. For example, the NTO weight value has no impact on the mean dose to the parotid glands for H&N cancers, whereas the mean dose to the heart decreases by more than 20% for esophageal treatment when the NTO is increased from 0.2 to 1000. However, some trends can be observed. The impact of NTO values up to 1 on doses is negligible for most OARs. For NTO values above 1, increasing the NTO value leads to an overall reduction in the OAR doses. Since the purpose of the NTO feature is to reduce the dose to healthy tissue, an important dosimetric index is the mean dose to the body-PTV. We observe that this index decreases significantly with an NTO value of 10. Averaged over all locations and patients, the mean dose to the body-PTV decreases by 0.9%, 5.9%, 13.5%, and 20.9% when the NTO increases from 0.2 to 1, 10, 100, and 1000, respectively.

The OAR doses decrease by approximately 1% for most OARs when the number of optimization rounds is increased from 3 to 6 (see Appendix D). The variation in the dosimetric indices is generally negligible when the number of optimization rounds is increased from 6 to 9.

### 4.5. Effect of Using a Ring on the NTO Weight

With VOLO^TM^ Classic, it is necessary to use optimization structures (rings), defined as being equal to the PTV plus a margin of several centimeters, to control the dose to healthy tissue areas not covered by the OARs. These rings should not be necessary with the NTO feature in VOLO^TM^ Ultra. Appendix E shows that the mean dose difference to the body-PTV between plans with and without a ring tends to decrease as the NTO increases. The difference decreases from 2.6% for an NTO of 0.2 to less than 1% for an NTO of 10. Therefore, we suggest that the plan does not need rings if a sufficiently high NTO weight value is used, which is on the order of 10 in our case. This is very important for the TD technique: the average dose to the body-PTV increases by 10% for the plan without a ring and an NTO weight of 0.2 compared with the plan with a ring and no NTO feature. The use of a ring had no significant impact on the BOT or PTV D_98%_/V_95%_ values, regardless of the NTO value (mean difference < 1%).

### 4.6. Effect of the LOT Cutoff Parameter on the Delivery Accuracy

**Figure 4:**
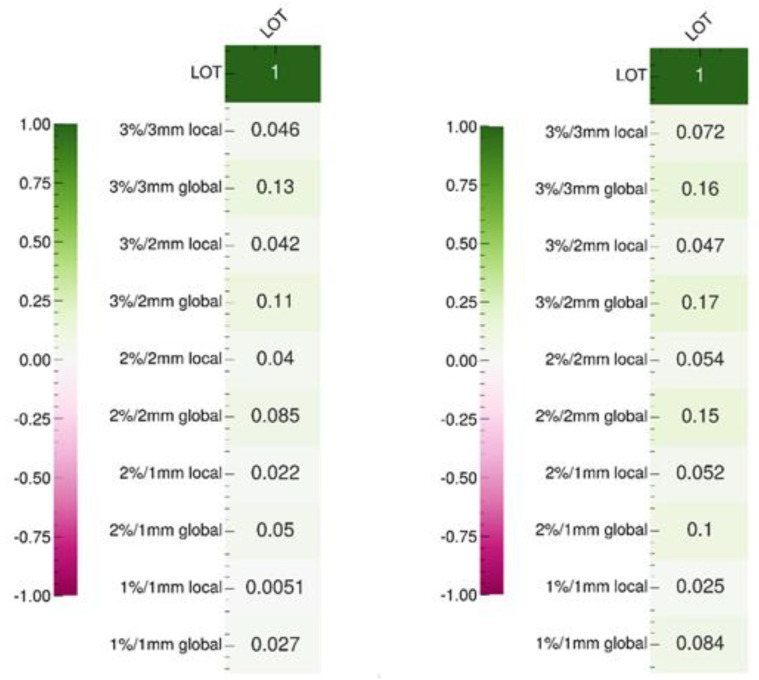
Pearson (**left**) and Spearman (**right**) correlation test results between the LOT cutoff and gamma index values according to the dose/distance criteria.

Figure 4 shows the Pearson’s and Spearman’s correlation test results based on the gamma indices measured for 36 treatment plans (Appendix C). The aim is to establish whether a linear or monotonic relationship exists between the LOT cutoff value and the gamma index results. The values of these two tests do not show that increasing the LOT cutoff value improves the delivery accuracy, regardless of the gamma index dose/distance criterion selected. Notably, although these results are not consistent with previous studies [8,9,15], a lack of correlation between LOT values and delivery accuracy has already been reported in the literature [16]. These seemingly contradictory observations may be explained by a relatively weak correlation, which is difficult to demonstrate and requires a larger sample size than used in our study. Another potential explanation is the insufficient impact of the LOT cutoff on the mean LOT value, which does not improve delivery accuracy.

We tested the correlations between the gamma index values of the 36 measured plans and 65 complexity metrics [12] (see Appendix F), as well as the correlations between these metrics and the LOT cutoff values (see Appendix G). Our results show that the LOT cutoff value is correlated with certain metrics that are derived based directly on LOT values, such as minLOT, as well as certain CLNS and CFNS scores. However, these metrics do not appear to be correlated with the gamma index values, which is consistent with our previous observations. Other metrics, such as sdFLOT, nOC, TA, and CLS, which are not correlated with the LOT cutoff value, appear to be correlated with the gamma index. We note that the number of evaluated plans is relatively small, as we measured 36 plans, compared with 881 measured plans in another study; thus, the test results should be considered carefully [13].

### 4.7. Synthesis and Suggested Planning Parameter Values

Table 4 summarizes the results obtained in this study. The table can be used to compare the magnitudes of the impacts of various parameters. Note that the table shows the average values for 36 patients and 6 locations and does not reflect interpatient and interlocation variations, which could be significant. Moreover, we note that these values partially depend on the weights applied to the targets and OARs. Thus, VOLO^TM^ Ultra users using this table should carry out evaluations based on their own patients to confirm the compatibility of their practices with the methods used in this study.

In VOLO^TM^ Ultra, the planner does not directly control the MF, as in VOLO^TM^ Classic; however, the planner can control the treatment time with the new AT feature. Increasing the AT value reduces the BOT at the expense of the PTV coverage and OAR dose. The user must, therefore, choose the best compromise according to the treatment priorities. However, there is a notable difference between the AT and MF: the BOT can be reduced by 50% when the MF is decreased from 3 to 1.5 [17], whereas we observe a maximum BOT reduction of approximately 27% when the AT is increased from 0 to 10. Therefore, the ability to modulate the BOT appears to be more limited with the AT than with the MF parameter. However, the maximum BOT can be manually limited to the desired time with VOLO^TM^ Ultra, which can reduce the BOT if needed.

Nevertheless, for the AT, our results show differences between locations treated with HT and TD plans. For TD plans, the AT value has no impact on the PTV coverage. Therefore, it seems preferable to systematically use an AT value of 10 since reducing this value increases the treatment time without any improvement in the target volume coverage and only a minor impact on the dose to the OARs.

Moreover, the results of this study showed no significant effect of the LOT cutoff on the delivery accuracy. We found that using an LOT value of 60 ms can reduce the target volume coverage, with marked effects for some patients. At this stage, and pending further studies, it therefore seems reasonable to limit the LOT value used in clinical practice to 40 ms.

As explained above, it is difficult to determine the NTO weight value, as the impact of this value depends on the weights applied to the OARs and targets. In our study and for our patients, we observed that an NTO value of approximately 10 is ideal. This value does not increase the BOT or reduce the PTV coverage, with a notable decrease in the dose to healthy tissue. For lower NTO values, the decrease in the dose to healthy tissue is minimal, while for higher NTO values, the BOT is increased, and the PTV coverage is significantly decreased. Finally, we observed that an NTO value of 10 eliminates the need for rings in the treatment plan.

Compared with the other optimization parameters, the number of optimization rounds has less impact on the time and dose metrics. There is a noticeable average variation in the metrics when the number of optimization rounds is changed from 3 to 6 (particularly in the dose to the OARs), and the metrics remain stable when the number of optimization rounds is changed from 6 to 9. Based on the calculation speed, a minimum of 6 rounds seems to be a good compromise, and the usefulness of more rounds should be assessed on a case-by-case basis.

### 4.8. Limitations and Perspectives

To our knowledge, this is the first study to assess the optimal VOLO^TM^ Ultra algorithm parameters for tomotherapy treatments. As previously mentioned, one limitation of this study is that some results depend on the weights applied to the targets and OARs, which are largely center/planner-specific. Therefore, the results of this article provide a baseline and reference understanding of the competing influences of the studied parameters rather than an absolute solution that can be generalizable to centers with different optimization practices. This is especially important for the determination of the optimal NTO weight value, which is input directly into the cost function of the optimization algorithm. This problem could be addressed by studying the impact of the NTO on the evaluation metrics as a function of the ratio between the weights of the organs at risk and NTO. It would also be interesting to study the NTO dose decay curve, as we did not vary this parameter.

Offset distance between PTV and gantry isocenter is known to influence the dosimetric quality and irradiation time of tomotherapy plans [18]. We did not include this parameter in our study, so its influence has yet to be investigated for Volo^TM^ Ultra.

The number of DQAs performed to test the usefulness of the new LOT cutoff functionality may seem limited. Although we were unable to demonstrate the impact of this parameter on the delivery accuracy, further investigations are still needed. Furthermore, concerning delivery accuracy, we focused only on the impact of LOT cutoff, but it would be interesting to study other parameters, such as the number of optimization rounds that influence each LOT to move the leaf efficiently.

## 5. Conclusions

In this study, we evaluated the effect of new optimization parameters in the^optimization^ VOLO^TM^ Ultra algorithm on HT and TD treatments. Optimal values for the AT, LOT cutoff, NTO weight, and number of optimization rounds were identified based on analyses of 1056 treatment plans created for 36 patients and six locations. For TD plans, we suggest systematically using an AT value of 10, while this value must be chosen depending on the patient for HT treatments. An NTO weight of approximately 10 appears to be ideal and eliminates the need to use rings in the treatment plan. A LOT cutoff value of 60 ms should be avoided since it seems to degrade dosimetry quality without any benefit on delivery accuracy. We recommend a minimum of 6 optimization rounds, as the usefulness of more rounds is not obvious. The result obtained in this study may be helpful to better understand VOLO^TM^ Ultra and to obtain better treatment plans.

## Figures and Tables

**Table 1:** Characteristics of the treatment plans by location.

Location	Number of Patients	Technique	FW/Pitch	Number of Fraction × Dose/Fraction
Whole breast (with nodes)	3 patients: left breast 3 patients: right breast	TomoHelical	5/[0.285–0.296]	3 patients: 25 × 2 Gy 3 patients: 15 × 2.67 Gy
Head-and-neck	6	TomoHelical	2.5/[0.428–0.440]	3 patients: 33 × 1.64–33 × 2.12 Gy (SIB) 3 patients: 33 × 1.65–33 × 2 Gy (SIB)
Cervix	6	TomoHelical	5/[0.431–0.435]	4 patients: 25 × 1.8 Gy 2 patients: 25 × 1.8–25 × 2.2 Gy (SIB)
Esophagus	6	TomoHelical	5/[0.427–0.443]	6 patients: 20 × 2 Gy
Hypofractionated	6	TomoHelical	5/[0.221–0.227]	6 patients: 2 × 6.5 Gy
Breast gland (without nodes)	3 patients: left breast 3 patients: right breast	TomoDirect	5/0.5	3 patients: 25 × 2 Gy 3 patients: 15 × 2.67 Gy

**Table 2:** Values of the optimization parameters were studied for each of the 36 patients. Bold: parameters that varied.

AT	LOT Cutoff (ms)	NTO Weight	Iteration Rounds	Use of a 5 cm Ring	Number of Plans
**0–2–4–6–8–10**	**20–40–60**	0.2	3 or 6 (for BG)	Yes	648
4 (for cervix, H&N, WB, and esophagus) or 10 (for BG and HP)	20	**0.2–1–10–100–1000**	3 or 6 (for BG)	No	180
4 (for H&N, WB, and esophagus) or 10 (for BG)	20	**0.2–1–10–100–1000**	3 or 6 (for BG)	Yes	120 ^a^
4 (for cervix, H&N, WB, and esophagus) or 10 (for BG and HP)	20	10	**3–6–9**	No	108

AT: accelerated treatment; BG: breast gland; H&N: head and neck; HP: hypofractionated palliative; LOT: leaf open/close time; NTO: normal tissue objective; WB: whole breast (with nodes). ^a^ Cervix and HP plans were not calculated using these optimization parameters.

**Table 3:** Description of the 36 plans for which delivery quality assurance was carried out.

Patients	Location/Treatment	AT	LOT Cutoff
1	Whole breast	0–4–10	20–40–60
2–3–4–5–6	Whole breast	4	20–40–60
7	Head and neck	4	20–40–60
8	Cervix	4	20–40–60
9	Esophagus	4	20–40–60
10	Hypofractionated	4	20–40–60

T: accelerated treatment; LOT: leaf open time.

**Table 4:** Summary of the impact of the AT, LOT cutoff, NTO weight, number of rounds, and use of rings on the BOT and PTV dosimetric metrics and OARs. The values reported in the table are averaged over 36 patients treated at 6 locations and are rounded to the nearest integer.

		BOT	PTV D_98%_/V_95%_	PTV D_2%_	OAR
AT	0->2	−11% (−7% for TD)	−2% (0% for TD)	≈ 0	+0 to 15% *
	2->4	−7% (−4% for TD)			
	4->6	−5% (−3% for TD)			
	6->8	−4% (−2% for TD)			
	8->10	−3% (−2% for TD)			
LOT cutoff	20->40	≈0	≈0	≈ 0	≈0
	40->60	−2%	−1%	+1%	−1% *
NTO	0.2->1	≈0	≈0	≈ 0	−1% **
	1->10	+2%	≈0	≈ 0	−5% **
	10->100	+12%	−2%	+1%	−8% **
	100->1000	+25%	−5%	+2%	−8% **
Rounds	3->6	≈0	≈0	≈ 0	−1%
	6->9	≈0	≈0	≈ 0	≈0
No rings		≈0	≈0	Not studied	+2% ** if NTO < 10 (+10% for TD) ≈0 ** if NTO ≥ 10

AT: accelerated treatment; BOT: beam-on time; LOT: leaf open-time; NTO: normal tissue optimization; OAR: organs at risk; PTV: planning target volume; TD: TomoDirect. * High standard deviation; depends on the patient and the OAR. ** Only for the body-PTV dose. ≈0 if less than 0.5%.

**Figure A1:**
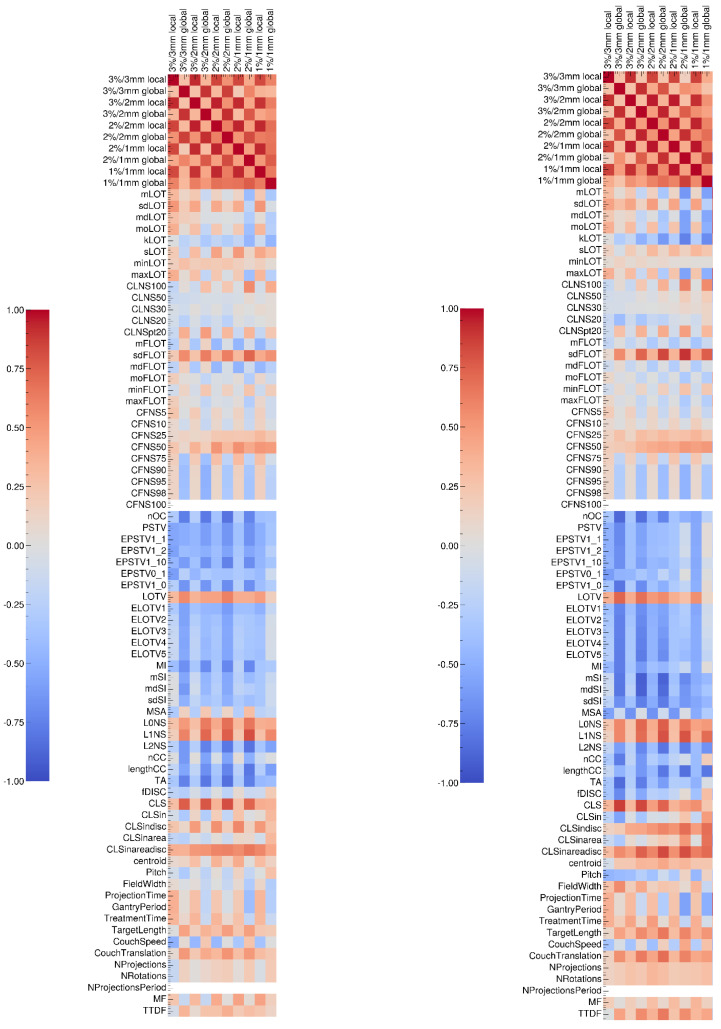
Sperman (**left**) and Pearson (**right**) correlation tests results between gamma-index values and complexity metrics specifically designed for tomotherapy treatments.

**Figure A2:**
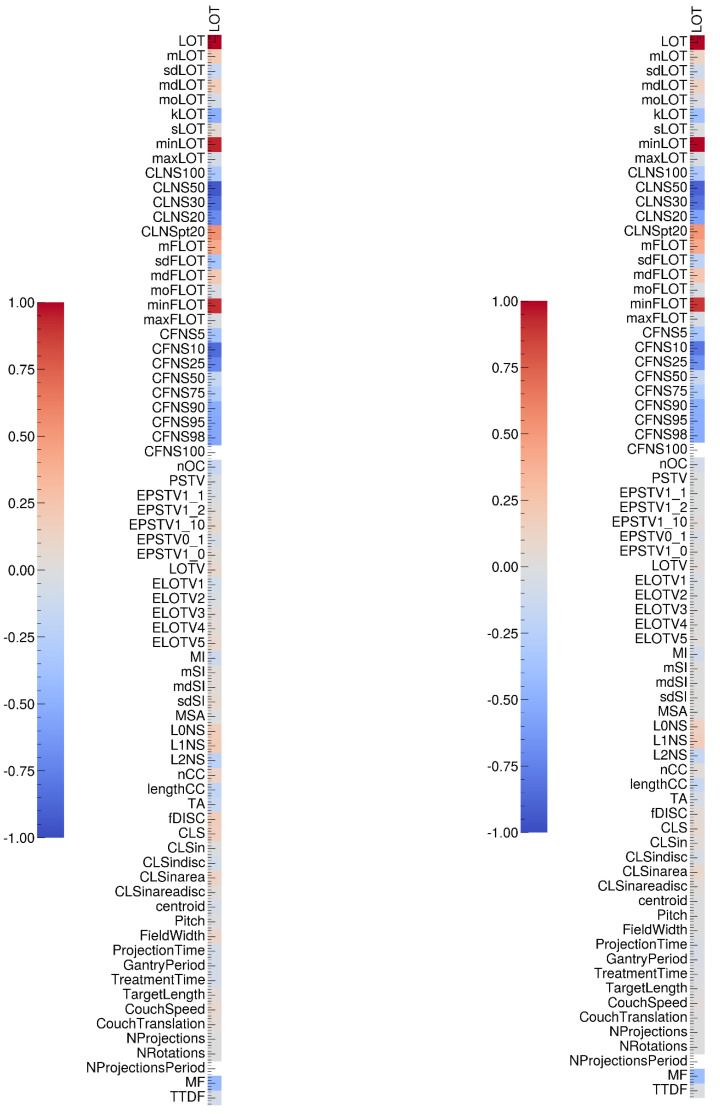
Sperman (**left**) and Pearson (**right**) correlation tests results between gamma-index values and complexity metrics specifically designed for tomotherapy treatments.

## Data Availability

The complete raw data are available in a data repository (https://data.mendeley.com/datasets/nz6ss5rgb5/draft?a=30c60a3c-b9d5-4bbc-84cc-c526a3cee215 (accessed on 1 May 2024)).

## Appendix A

Constraints used for the two breast patients in this study (one for the whole breast and one for the breast gland).

**Whole breast**

Prescription = 50.00 Gy, 50% of PTVL_50

Delivery Mode = Helical

Plan Mode = IMRT

Jaw Mode = Dynamic

Fractions = 25

Pitch = 0.289

Synchrony Method = None

Final Dose Resolution = High

Auto Beam On Time Enabled = Yes

Accelerate Treatment = 4

Optimization Resolution = Low

Leaf Open/Close Time Cut-Off (ms) = 20

Accelerate Optimization = 1

Min # Iteration Rounds = 3

[Target Goals]

 PTVNL_50

  [DVH Goals]

   Goal Type = Min

   Weight = 180.0

   Specified Dose (Gy) = 50.00

   Volume (%) = 100.0

   Goal Type = Max

   Weight = 70.0

   Specified Dose (Gy) = 50.00

   Volume (%) = 0.0

 PTVL_50

  [DVH Goals]

   Goal Type = Min

   Weight = 100.0

   Specified Dose (Gy) = 50.00

   Volume (%) = 100.0

   Goal Type = Max

   Weight = 70.0

   Specified Dose (Gy) = 50.00

   Volume (%) = 0.0

[Critical Goals]

 Heart

  [DVH Goals]

   Goal Type = Max

   Weight = 7.0

   Specified Dose (Gy) = 50.00

   Volume (%) = 0.0

   Goal Type = Mean

   Weight = 5.0

   Specified Dose (Gy) = 3.00

 Lung_R

  [DVH Goals]

   Goal Type = Mean

   Weight = 5.0

   Specified Dose (Gy) = 1.00

 Lung_L

  [DVH Goals]

   Goal Type = Max

   Weight = 7.0

   Specified Dose (Gy) = 50.00

   Volume (%) = 0.0

   Goal Type = Mean

   Weight = 15.0

   Specified Dose (Gy) = 1.00

 Breast_R

  [DVH Goals]

   Goal Type = Max

   Weight = 4.0

   Specified Dose (Gy) = 20.00

   Volume (%) = 0.0

   Goal Type = Mean

   Weight = 4.0

   Specified Dose (Gy) = 5.00

 Esophagus

  [DVH Goals]

   Goal Type = Mean

   Weight = 4.0

   Specified Dose (Gy) = 8.00

 SpinalCanal

  [DVH Goals]

   Goal Type = Max

   Weight = 4.0

   Specified Dose (Gy) = 20.00

   Volume (%) = 0.0

 Hum_Head_PRV_L

  [DVH Goals]

   Goal Type = Max

   Weight = 3.0

   Specified Dose (Gy) = 20.00

   Volume (%) = 1.0

 Trachea

  [DVH Goals]

   Goal Type = Max

   Weight = 3.0

   Specified Dose (Gy) = 20.00

   Volume (%) = 1.0

 Thyroid_Glnd

  [DVH Goals]

   Goal Type = Max

   Weight = 3.0

   Specified Dose (Gy) = 20.00

   Volume (%) = 1.0

 _optiChauffeBloc

  [DVH Goals]

   Goal Type = Max

   Weight = 2.0

   Specified Dose (Gy) = 36.00

   Volume (%) = 1.0

 _optiSusclav

  [DVH Goals]

   Goal Type = Max

   Weight = 2.0

   Specified Dose (Gy) = 36.00

   Volume (%) = 1.0

 Liver

  [DVH Goals]

   Goal Type = Mean

   Weight = 4.0

   Specified Dose (Gy) = 1.00

 _optiAbdomen

  [DVH Goals]

   Goal Type = Mean

   Weight = 2.0

   Specified Dose (Gy) = 1.00

[Beam Intersection]

 _OptiLiver: Exit Only

 _blocD: Exit Only

 _blocG: Exit Only

 _optiHeart: Exit Only

 _optiLL: Exit Only

 _optiLR: Exit Only

**Breast gland**

Prescription = 40.00 Gy, 50% of PTVR_40_seinD

Delivery Mode = Direct

Plan Mode = IMRT

Jaw Mode = Dynamic

Fractions = 15

Pitch = 0.5

Synchrony Method = None

Final Dose Resolution = High

Auto Beam On Time Enabled = Yes

Accelerate Treatment = 8

Optimization Resolution = Medium

Leaf Open/Close Time Cut-Off (ms) = 60

Accelerate Optimization = 1

Min # Iteration Rounds = 6

[Beam Angles]

 TGI1 Angle = 56.00 −Xg (leaves) = 3 +Xg (leaves) = 0

  Target VOIs: PTVR_40_seinD

 TGI2 Angle = 59.00 −Xg (leaves) = 3 +Xg (leaves) = 0

  Target VOIs: PTVR_40_seinD

 TGE1 Angle = 239.00 −Xg (leaves) = 0 +Xg (leaves) = 3

  Target VOIs: PTVR_40_seinD

 TGE2 Angle = 243.00 −Xg (leaves) = 0 +Xg (leaves) = 3

  Target VOIs: PTVR_40_seinD

[Target Goals]

 PTVR_40_seinD

  [DVH Goals]

   Goal Type = Min

   Weight = 100.0

   Specified Dose (Gy) = 40.00

   Volume (%) = 100.0

   Goal Type = Max

   Weight = 100.0

   Specified Dose (Gy) = 40.00

   Volume (%) = 0.0

[Critical Goals]

 Heart

  [DVH Goals]

   Goal Type = Mean

   Weight = 10.0

   Specified Dose (Gy) = 1.00

   Goal Type = Max

   Weight = 10.0

   Specified Dose (Gy) = 36.00

   Volume (%) = 0.0

 Lung_R

  [DVH Goals]

   Goal Type = Mean

   Weight = 10.0

   Specified Dose (Gy) = 1.00

   Goal Type = Max

   Weight = 10.0

   Specified Dose (Gy) = 36.00

   Volume (%) = 0.0

 Liver

  [DVH Goals]

   Goal Type = Max

   Weight = 5.0

   Specified Dose (Gy) = 39.00

   Volume (%) = 0.0

   Goal Type = Max

   Weight = 5.0

   Specified Dose (Gy) = 10.00

   Volume (%) = 10.0

 Breast_L

  [DVH Goals]

   Goal Type = Max

   Weight = 10.0

   Specified Dose (Gy) = 2.00

   Volume (%) = 0.0

 ring5-ptv

  [DVH Goals]

   Goal Type = Max

   Weight = 5.0

   Specified Dose (Gy) = 39.00

   Volume (%) = 0.0

   Goal Type = Max

   Weight = 5.0

   Specified Dose (Gy) = 16.00

   Volume (%) = 10.0

 opti2

  [DVH Goals]

   Goal Type = Max

   Weight = 5.0

   Specified Dose (Gy) = 38.00

   Volume (%) = 0.0

 opti3

  [DVH Goals]

   Goal Type = Max

   Weight = 5.0

   Specified Dose (Gy) = 39.00

   Volume (%) = 0.0

[Beam Intersection]

 Breast_L: Exit Only

## Appendix B

**Table A1:** Dosimetric indices recorded for organs at risk, according to localization.

Localization		OAR	Dosimetric Parameter
Whole Breast (with nodes)		Heart, ipsilateral lung	Dmean (Gy), V_20_ or V1_7Gy_ (%) ^a^
		Controlateral lung and breast, esophagus, spinal canal, body-PTV	Dmean (Gy)
Head-and-neck		Brainstem, spinalcord	Dmax (Gy)
		Left and right parotids, larynx, constrictor muscle, oral cavity, body-PTV	Dmean (Gy)
Cervix		Bowel_Bag	V_20_ and V_45Gy_ (cm^3^)
		Bladder, rectum anal canal, body-PTV	Dmean (Gy)
Esophagus		Lungs-PTV	Dmean (Gy), V_20_ and V_30Gy_ (%)
		Heart	Dmean (Gy) and V_30Gy_ (%)
		Liver	V_15Gy_ (cm^3^)
		SpinalCanal, body-PTV	Dmax (Gy)
Palliative	Stomach (3 patients)	Kidneys, liver, heart, body-PTV	Dmean (Gy)
	Rectum	Bladder, bowel bag, body-PTV	Dmean (Gy)
	Cervix	Rectum, bladder, bowel bag, body-PTV	Dmean (Gy)
	Nasal cavity	Left and right eyes, oral cavity, body-PTV	Dmean (Gy)
Breast gland (without nodes)		Heart	Dmean (Gy)
		Heart, ipsilateral lung	V_20_ or V_17Gy_ (%) ^a^
		Controlateral lung	V_4_ or V_3.5Gy_ (%) ^a^
		Body-PTV	Dmean (Gy)

Dmean: mean dose delivered to the organ; OAR: organ at risk; PTV: planning target volume; VxGy: part of the organ receiving more than x Gy, in % or cm^3^ of the organ. ^a^ depending on the dose prescribed to the PTV.

## Appendix C

**Table A2:** Gamma-index values (%) measured for 36 treatment plan configurations for different dose/distance.

			3%/3 mm 10% Local			3%/3 mm 10% Global			2%/2 mm 10% Local			2%/2 mm 10% Global			1%/1 mm 10% Local		
Patient	Localization	AT	LOT 20	LOT 40	LOT 60	LOT 20	LOT 40	LOT 60	LOT 20	LOT 40	LOT 60	LOT 20	LOT 40	LOT 60	LOT 20	LOT 40	LOT 60
1	Whole Breast	AT 0	91	91.6	92	99.4	99.1	99.5	73.2	73.5	74.4	95.1	95.4	96.5	45.1	44.5	45.4
1	Whole Breast	AT 4	91.8	91.4	93	99.4	99.7	99.7	74	71.3	74	95.6	96.2	96.4	45.2	42.5	44.8
1	Whole Breast	AT 10	92.5	91.5	92.3	99.7	99.7	99.7	74.5	74.1	74.2	96.4	96.6	97.8	43.9	43.3	44.7
2	Whole Breast	AT 4	92.1	93	92	98.3	98.4	98.4	76.5	77.3	77.5	93.3	93.2	93.5	46.4	45	45.8
3	Whole Breast	AT 4	96	95.7	96.6	99.5	99.5	99.8	84.9	85.4	86.8	95.1	95.7	96.9	54.4	53.8	54.7
4	Whole Breast	AT 4	89.6	89.1	89.3	98.8	99	99.1	72.9	72.6	74.7	92	92.9	92.6	42.9	42.8	45.4
5	Whole Breast	AT 4	86.6	85.7	85.2	99.8	99.6	99.8	69.3	66.2	66.1	95.2	94.6	95	38.4	35.6	35.3
6	Whole Breast	AT 4	93.5	93.8	93.9	100	99.5	100	81.5	82.2	81.6	96.9	96.4	96.7	48.4	46.6	46.7
7	Head and neck	AT 4	88	88.5	88.7	96.7	97.1	97.5	68.5	70.5	71.6	89.1	89.2	90	36	35.5	37.5
8	Cervix	AT 4	87.7	88.8	90.2	96.8	97.5	98.2	66.9	67.2	66.8	88.5	90	89.4	38.5	38.6	36.6
9	Esophagus	AT 4	88.1	88.1	87.5	99.2	99.6	99.6	66.8	65.7	67.7	92.2	93.3	93.8	36.8	37.2	38.2
10	Paliative hypofrac.	AT 4	92	91.3	91.9	97.9	97.5	97.9	68.1	66.9	68.6	81.8	80.9	83.1	37.4	36.8	39.1
			1%/1 mm 10% global			3%/2 mm 10% local			3%/2 mm 10% global			2%/1 mm 10% local			2%/1 mm 10% global		
1	Whole Breast	AT 0	63.1	63.7	64.8	80	79.3	80.7	98.6	98.4	98.7	50.2	50.2	50.3	88.6	89.1	90.4
1	Whole Breast	AT 4	67.2	62.1	66.6	82	79.6	82.4	98.9	99.2	98.9	49.3	47.5	50.4	90.9	90.5	91.1
1	Whole Breast	AT 10	64.2	64.6	66.2	82.7	82.6	83.9	99.4	99.4	99.5	49.8	49.1	51.2	90.3	91.1	93
2	Whole Breast	AT 4	57.4	56.2	57.5	84.7	85.6	85.2	97.4	97.7	98	54	53.2	55.4	83	82.9	83.2
3	Whole Breast	AT 4	69.7	68.8	69.3	91.6	91.2	92.2	98.6	98.6	99.4	63.8	63.7	64.8	87.6	88.4	88.8
4	Whole Breast	AT 4	56.1	54.6	57.5	80.9	80.5	81.4	97.5	97.7	98	50.9	49.2	51.7	83.8	84.2	85
5	Whole Breast	AT 4	56.7	55	54.6	76.1	73.5	74.2	98.5	98.5	98.8	43.5	41.3	41.5	85.2	84.5	85.3
6	Whole Breast	AT 4	65.5	66.3	65	87.1	88.2	87.7	98.9	98.4	98.7	56.6	55	55.3	88.5	87.9	88
7	Head and neck	AT 4	63.2	63.5	64.7	77.9	77.9	79.5	94.3	94.8	95.1	44.7	45.5	45.7	83.1	82.1	83.8
8	Cervix	AT 4	62.9	62.8	63.6	77.2	78.4	78.5	93.8	95.3	95.4	47.5	46.9	45.8	84.6	85.7	85.4
9	Esophagus	AT 4	48.8	49.1	50.2	74.3	73.9	75.3	98.2	98.6	98.7	42.1	42	43.2	80.8	82.3	82.9
10	Paliative hypofrac.	AT 4	40.8	40	41.8	80.2	78.6	79.7	93.3	92.5	94.3	44	43.8	45	64.5	61.7	64.4

## Appendix D

Impact of variations in the number of optimizations rounds on the OAR dosimetric indices. Not all OARs are reported for clarity. The values are averaged over the six patients for each location. The AT variation values are averaged over the three LOT values, and the LOT variation values are averaged over the six AT values. The values are normalized to the first value of each parameter studied to analyze the variation in the parameters. The error bars represent one standard deviation.

## Appendix E

Mean deviation between the treatment plans calculated with and without the 5 cm ring as a function of the NTO weight value for the BOT, PTV D_98%_ or V_95%_ and body-PTV mean dose. The error bars represent one standard deviation.
